# Immunological and Toxicological Considerations for the Design of Liposomes

**DOI:** 10.3390/nano10020190

**Published:** 2020-01-22

**Authors:** Collin T. Inglut, Aaron J. Sorrin, Thilinie Kuruppu, Shruti Vig, Julia Cicalo, Haroon Ahmad, Huang-Chiao Huang

**Affiliations:** 1 Fischell Department of Bioengineering, University of Maryland, College Park, MD 20742, USAtkuruppu@terpmail.umd.edu (T.K.); svig@umd.edu (S.V.); jcicalo@terpmail.umd.edu (J.C.); 2 Marlene and Stewart Greenebaum Cancer Center, University of Maryland School of Medicine, Baltimore, MD 21201, USA; HAhmad@som.umaryland.edu; 3 Department of Neurology, University of Maryland School of Medicine, Baltimore, MD 21201, USA

**Keywords:** liposomes, toxicity, immunomodulation, cancer, gene and drug delivery

## Abstract

Liposomes hold great potential as gene and drug delivery vehicles due to their biocompatibility and modular properties, coupled with the major advantage of attenuating the risk of systemic toxicity from the encapsulated therapeutic agent. Decades of research have been dedicated to studying and optimizing liposomal formulations for a variety of medical applications, ranging from cancer therapeutics to analgesics. Some effort has also been made to elucidate the toxicities and immune responses that these drug formulations may elicit. Notably, intravenously injected liposomes can interact with plasma proteins, leading to opsonization, thereby altering the healthy cells they come into contact with during circulation and removal. Additionally, due to the pharmacokinetics of liposomes in circulation, drugs can end up sequestered in organs of the mononuclear phagocyte system, affecting liver and spleen function. Importantly, liposomal agents can also stimulate or suppress the immune system depending on their physiochemical properties, such as size, lipid composition, pegylation, and surface charge. Despite the surge in the clinical use of liposomal agents since 1995, there are still several drawbacks that limit their range of applications. This review presents a focused analysis of these limitations, with an emphasis on toxicity to healthy tissues and unfavorable immune responses, to shed light on key considerations that should be factored into the design and clinical use of liposomal formulations.

## 1. Introduction

Liposomes are vesicular structures composed of one or more concentric lipid bilayers surrounding an aqueous cavity [1,2,3,4]. The bilayers are predominantly composed of phospholipids, where the polar head groups interface with the outer and inner aqueous phases and the hydrophilic tails are sequestered within the bilayer [4]. Since their discovery by Alec Bangham in the 1960s [5,6], liposomes have been studied extensively as drug delivery vehicles due to their capacity to load both hydrophilic and hydrophobic agents, as well as their high biocompatibility and tunable size, charge, and surface properties [1,2,3,4]. Liposomal encapsulated drugs first reached the clinic in 1995 with the US Food and Drug Administration (FDA)-approval of Doxil (liposomal doxorubicin) for the treatment of AIDS-related Kaposi′s sarcoma, and Doxil was later approved to treat ovarian cancer and multiple myeloma [7]. Since the FDA approval of Doxil, numerous liposomal formulations have been employed in the clinic for a wide array of applications, including cancer therapeutics, fungal disease treatment, analgesics, photodynamic therapy, and viral vaccine delivery [8,9]. However, despite the increasing prominence of liposomal drugs in the clinic, there is still limited knowledge regarding their toxicological effects on healthy cells and tissues, as well as the immunological responses they can elicit.

Phospholipids, the primary building blocks of liposomes, are amphipathic molecules, meaning they have a hydrophilic region (e.g., polar phosphate head) and a hydrophobic section (e.g., non-polar fatty acid tail). When hydrated in an aqueous solution under artificial conditions, phospholipids spontaneously organize into liposomes due to their thermodynamic phase properties and self-assembling characteristics [10]. The physio-biochemical characteristics of liposomes can be modified by altering the types and ratios of phospholipids, as well as incorporating cholesterol into the bilayer and decorating the liposomal surface with polyethylene glycol (PEG). These modifications can have drastic effects on healthy cells and tissues, as well as activate or suppress the immune system. These complex interactions therefore have immense implications for the clinical use of liposomal formulations and will be discussed in depth later in this review. Extensive research has been done to develop a variety of techniques to achieve optimized liposome formation and drug loading. Incorporating therapeutic agents into liposomes can be achieved either during liposome formation (e.g., passive loading) or after liposome formation (e.g., active loading). Passive loading can be further divided into three categories: mechanical dispersion methods, solvent dispersion methods, and detergent removal methods [2,11,12]. Alternatively, active loading can be accomplished by establishing a pH gradient, causing the unionized drugs that penetrate the lipid bilayer to become ionized due to the low pH within the liposome, resulting in entrapment [13,14]. FDA approval has been granted for both passively loaded liposomal agents (e.g., Visudyne® and AmBisome®) and actively loaded liposomal agents (e.g., Doxil, Myocet®, and Onivyde®) [15]. Most of the clinically used liposome-based products are administered by intravenous (IV) injection, though some are also given by intramuscular injection (e.g., Inflexal® V and Epaxal®), by epidural injection (e.g., DepoDur™), or by intrathecal injection (e.g., Depocyt®) [8].

Liposomes are particularly useful for delivering hydrophobic agents, which otherwise have poor solubility in aqueous solutions and limited bioavailability [16,17]. Verteporfin (also known as benzoporphyrin derivative), for example, is a hydrophobic photosensitizer that is used for photodynamic therapy, a light-based therapeutic modality. While verteporfin self-aggregates in aqueous solutions, liposomal verteporfin (marketed as Visudyne®) has improved solubility for IV administration and is FDA-approved to treat wet age-related macular degeneration [18,19]. To date, Visudyne® is being evaluated in multiple clinical trials for photodynamic therapy of cancer due in part to its favorable pharmacokinetic profiles (i.e., rapid clearance), leading to low phototoxic skin reactions [20]. In addition to improving drug solubility, liposomes have a variety of other advantages as drug delivery vehicles, such as high biocompatibility, modifiable physicochemical and biophysical properties, and protection of large drug payloads from degradation, inactivation, and dilution in circulation [3,21]. Liposomes are also being engineered to deliver drugs to parts of the body that are exceptionally difficult to access (e.g., central nervous system) [22]. Intravenously-injected liposomal drug formulations provide the additional benefit of preferentially accumulating in tumor tissues due to the enhanced permeability and retention (EPR) effect, thereby incorporating an inherent mechanism for passive tumor targeting [23,24].

Another key advantage is that liposomal encapsulation of drugs increase the therapeutic index by decreasing the accumulation of drugs in organs and other healthy tissues [23,24,25]. For example, anthracyclines, a group of potent chemotherapeutics, can induce severe cardiotoxicities, including cardiomyopathy and congestive heart failure. This limits the range of doses that can be safely administered. Using liposomes to deliver doxorubicin, one of the most widely used and most cardiotoxic anthracyclines, avoids drug accumulation in myocardial tissues, improving the overall response rates and attenuating the risk of cardiotoxicity [26,27]. For instance, liposomal encapsulation of doxorubicin increased the median lethal dose (LD_50_) by approximately 2-fold in mice (32 vs. 17 mg/kg) and 1.5-fold in beagle dogs (2.25 vs. 1.5 mg/kg) compared to free form doxorubicin [28]. Similarly, liposomal encapsulation of irinotecan (i.e., Onivyde®), another chemotherapeutic agent used in multiple solid organ cancers, enables improved pharmacokinetics, increased maximum tolerable dose, and protection of irinotecan from hydrolysis-mediated inactivation in circulation [29]. Though liposomes have demonstrated the ability to reduce the accumulation of drugs in some organs and healthy tissues, they tend to reroute them to other sites. A meta-analysis concluded that on average, less than 1% of nanoparticles end up delivered to solid tumors [30]. This underscores a key problem for liposomal agents, which largely accumulate in organs associated with the mononuclear phagocyte system (MPS), where they can impair liver function by attenuating phagocytic capacity and depleting macrophages [31].

**Figure 1:**
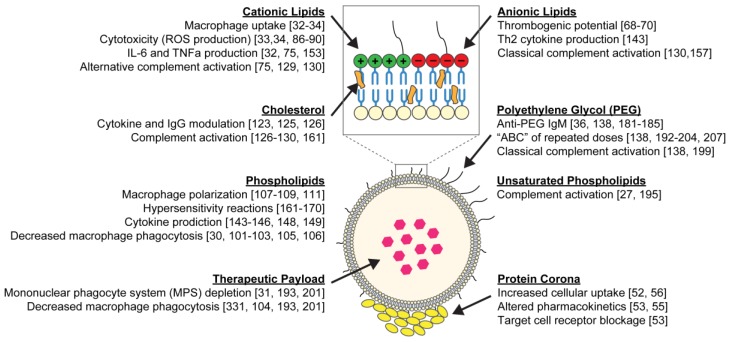
Schematic of a liposome and its common building blocks. The liposomal bilayer is composed of phospholipids (neutral, cationic or anionic) and cholesterol, and the surface can be decorated with polyethylene glycol (PEG). Hydrophilic drugs can be entrapped within the aqueous core, while hydrophobic drugs can be loaded into the lipid bilayer. Once injected into the bloodstream, a protein corona, comprised largely of apolipoproteins, immunoglobulins, and complement proteins, is formed on the liposome surface. The protein corona, which is impacted by the liposomal surface chemistry, governs liposome–cell interactions. The immunological and toxicological effects caused by each liposome component, imparted on cells throughout the body, are summarized throughout the diagram.

While liposomes are used to reduce systemic toxicity from the encapsulated agents, the liposome itself can impart toxicity to normal tissues and elicit an immune response (Figure 1). Cationic liposomes, which are largely studied for gene delivery, are known to elicit toxicity in macrophages, macrophage-like cells, and monocyte-like cells, as well as alter the secretion of important immuno-modulators [32,33,34]. Liposomes can also trigger immunogenic responses, though the nature and degree of these responses can vary depending on several properties, including surface charge, size, and pegylation [35,36,37]. Therefore, despite the many advantages of using liposomes for drug delivery, there are also important drawbacks that must be considered, as they present real challenges for clinical translation. This mini-review will explore these limitations in-depth, with an emphasis on toxicities and unfavorable interactions with the immune system, to shed light on some of the lesser-known areas where liposomes have room for improvement as drug delivery vehicles.

## 2. Toxicological Evaluation of Liposomes and Their Building Blocks

Liposomes are typically composed of numerous building blocks, including phospholipids, cholesterol, and PEG, each impacting their functionality. Phospholipids, the main component of liposomes, are diverse molecules with a variety of isomers. The most commonly used phospholipids for liposome formation include lecithins from egg and soy, as well as sphingomyelins [38]. Common variations to the phospholipid hydrophobic acyl (fatty acid) tail include the degree of saturation (i.e., saturated vs. unsaturated) and length, which can impact the oxidizing properties and drug release profiles of liposomes [39]. For example, Luo et al. showed that photodynamic therapy-mediated oxidation of unsaturated lipids (i.e., 1,2-dioleoyl-sn-glycero-3-phosphocholine, or DOPC, 18:1 PC) in the liposomal bilayer could facilitate the controlled release of doxorubicin. Increasing the concentration of DOPC in liposomes from 0% to 10% enhanced the release rate of doxorubicin from liposomes by up to 10-fold upon light-activation of photosensitizers [40]. Different phospholipid head groups, with some of the most prevalent being phosphatidylcholine (PC), phosphatidylserine (PS), and phosphatidylethanolamine (PE), can alter the surface charge and the polymorphic phase (i.e., the type of assembly) in an aqueous medium [41]. Although PC is a neutral molecule, it has a positively charged choline group attached to a negatively charged phosphate group, making it a zwitterionic molecule. Alternatively, PS contains a negatively charged phosphate group attached to the serine group, giving it an overall negative charge, mainly due to the low (2.2–3.6) pKa of the carboxyl group within the serine [42]. More recently, there is increased research interest in formulations of lipid–drug (or photosensitizer) conjugates to improve the pharmacokinetics and therapeutic index of liposomal therapeutics [43,44,45,46,47]. Similarly, the incorporation of PEG, a polymeric steric stabilizer, is widely used to increase the blood circulation time of liposomal formulations [48,49]. The addition of PEG into liposomal doxorubicin extended the terminal half-life from 16.4 h to 77 h in patients with metastatic breast cancer [50,51]. Once liposomes enter the bloodstream, plasma proteins interact with and adsorb onto the liposome surface. It has been previously shown that the liposomal surface chemistry (e.g., charge and pegylation) impacts the blood protein adsorption (known as the “protein corona”), which influences how liposomes interact with healthy and diseased cells [52,53,54]. Protein adsorption occurs due to an advantageous increase in entropy, resulting in a decrease in Gibbs free energy (ΔG_abs_) [55]. A comprehensive study by Hadjidemetriou et al. examined the in vitro and in vivo protein corona formation on three different types of liposomes, including pegylated liposomes, non-pegylated liposomes, and monoclonal antibody-conjugated liposomes [56]. In this study, pegylated liposomes had the lowest protein adsorption at ~10,000 μg protein/μM lipid, while non-pegylated liposomes had double the amount of protein adsorption. However, all liposomes had relatively consistent protein corona compositions, with the most abundant bound proteins being apolipoproteins, immunoglobulins, and complement proteins.

While the topic of nanoparticle-protein adsorption is an active field of study [55,56,57,58], several studies have evaluated the toxicity and side effects of liposomes. Papahadjopoulos et al. first reported the cytotoxicity of liposomes in healthy mouse fibroblast L929 cells in 1973. They discovered that incorporation of 3% or 10% of lysophosphatidylcholine (lysolecithin, lysoPC) into liposomes increases cellular uptake (i.e., polykaryocytosis) by ~7%. The number of viable L929 cells after liposomal incubation was significantly lowered from 97% to less than 37% [59]. Similarly, Pagano et al. showed that extensive accumulation of egg yolk lecithin vesicles in Chinese hamster lung fibroblast V79 cells result in considerable cell death [60]. One of the first studies evaluating liposomal toxicity in vivo was carried out by Adams and colleagues in 1977 [61]. In this study, five different types of liposomes with varying net surface charges were synthesized and injected into the mouse brain. Results showed that liposomes containing 9 mol% of dicetyl phosphate (net negative charge) were the most toxic, causing epileptic seizures and rapid death in animals. Injection of dicetyl phosphate alone resulted in the death of all mice within one hour. Brain sections stained with haematoxylin and eosin (H&E) revealed tissue necrosis, nerve damage, and loss of Nissl substance. Similarly, liposomes containing 9 mol% of stearylamine (net positive charge) caused widespread brain damage and respiratory failure. Alternatively, liposomes containing 9 mol% of phosphatidic acid (net negative charge) were well tolerated with only small hemorrhages and necrosis limited to the injection site. The least toxic liposomes were those composed of 45 mol% of lecithin or dipalmitoyl lecithin (neutral charge), which resulted in minimal toxic reactions and morphological changes, even after three repeated doses. While liposomes are typically considered pharmacologically inactive with minimal toxicity [2,21], their toxicity is tightly related to the type of model, exposure time, dose, and/or surface properties. For example, Shi et al. showed the median lethal concentration (LC_50_) using F98 glioma cells was 6.07 μM for empty DPPC-based cationic liposomes while DSPC-based anionic liposomes had an LC_50_ > 509 μM. Within an intracranial F98 glioma rat model, carboplatin loaded cationic pegylated liposomes resulted in a median survival time of 29 days, while anionic pegylated liposomes had a median survival of 49.5 days [62]. Similarly, Fan et al. showed that cationic liposomes incubated with bone marrow-derived dendritic cells had an LC_50_ around 0.2 mg/mL, while ovalbumin-loaded anionic liposomes had an LC_50_ > 4 mg/mL [63]. In Section 2, we examine some of the different mechanisms through which liposomes induce cellular toxicity.

As liposomes rose in popularity, many research groups began to modify the phospholipid structure and test different surface chemistries [64,65]. Studies in the early 1990s by Parnham et al. [66] and Lappalainen et al. [67] examined and suggested different assays for the screening of liposomal toxicity. Some of these in vitro viability and morphology assays include 3-(4,5-dimethylthiazol-2-yl)-2,5-diphenyltetrazolium bromide (MTT), 3H thymidine uptake, and lactate dehydrogenase (LDH). It was shown that 40 μM of liposomes containing dimethyldioctadecyl ammonium bromide (DDAB, 18:0) and dioleoylphosphatidylethanolamine (DOPE, 18:1 PE) reduce the viability of human cervical squamous cell carcinoma (CaSki) cells by 50% [67]. Liposomes containing N-(2,3-Dioleoyloxy-1-propyl)trimethylammonium methyl sulfate (DOTAP, 18:1 TAP) at a concentration of 10 μM or 40 μM only had minor effects on the CaSki cells [67]. There is also evidence demonstrating that anionic lipids have a higher thrombogenic potential [68]. Anionic liposomes have been shown to induce platelet aggregation in guinea pigs [69], as well as blood cell aggregation in mice, which became temporally trapped in lung capillaries [70]. To ensure that IV injection of liposomes does not induce pro-aggregatory effects or pharmacodynamic changes (e.g., necrosis, edema, loss of tissue) at the site of entry, appropriate platelet aggregation assays and local tolerance studies should be performed. The mechanical damage during injection should be accounted for as well, and distinguished from any toxicological effects [66]. In April 2018, the FDA published updated guidelines for liposome products in industry [71]. Although explicit details are not specified, critical parameters ranging from physicochemical characterization (e.g., pegylation, net charge, the leakage rate during shelf storage) to pharmacokinetic profiles are established. Radiolabeled mass-balance excretion and metabolism studies are required, as they reveal quantitative connections to toxicity, and provide information on the in vivo activity of new chemical entities including pharmacology and pharmacokinetics [72].

### 2.1. Toxicity of Cationic Liposomes

Cationic liposomes have been studied extensively to enhance the stability and delivery of negatively charged nucleic acid therapeutics [73,74,75,76,77,78], and their toxicities and side effects have also been carefully examined [32,33,34,79,80,81,82,83,84,85,86]. For example, Filion and Phillips studied the toxicity and immunomodulation of seven different DOPE (18:1 PE)-based cationic liposomes that range from +15.0 to +42.9 mV [32]. In this comprehensive study, liposomes containing DOTAP, DDAB, 1,2-dimyristoyl-3-trimethylammonium-propane (DMTAP, 14:0 TAP), 1,2-dipalmitoyl-3-trimethylammonium-propane (DPTAP, 16:0 TAP), or 1,2-stearoyl-3-trimethylammonium-propane (DSTAP, 18:0 TAP) were highly toxic to macrophages and monocyte-like U937 cells, but not to resting or activated T lymphocytes. It was speculated that these cationic liposomes were non-toxic to T cells due, in part, to minimal uptake. Additionally, the cytotoxicity of cationic liposomes increased at higher zeta potentials. DDAB-containing liposomes (+40.0 ± 9.0 mV) were the most toxic to macrophages, with a 50% effective dose (ED_50_) <10 nmol/mL, while DSTAP-containing liposomes (+15.0 ± 6.3 mV) were the least toxic, with an ED_50_ >1000 nmol/mL. Loading plasmid DNA into liposomes significantly reduced the zeta potential but did not decrease their cytotoxicity [32]. The cytotoxicity of the liposomes was significantly lowered by incorporating 10 mol% of 1,2-dipalmitoyl-sn-glycero-3-phosphoethanolamine (DPPE, 16:0 PE)-PEG2000 or replacing DOPE with 1,2-dipalmitoyl-sn-glycero-3-phosphocholine (DPPC, 16:0 PC). These modifications reduced the binding and endocytosis of liposomes by ~96% by macrophages and hindered their ability to destabilize the endosomal membrane [32]. In addition to inducing cytotoxicity in macrophages, the cationic liposomes also mitigated the secretion of nitric oxide (NO) and tumor necrosis factor-α (TNF-α), which are important signaling molecules for triggering non-specific defense against pathogens and enhancing T and B lymphocyte responsiveness [32].

Similarly, Takano et al. and Aramaki et al. showed that the intracellular uptake of cationic liposomes (~42 mV) in mouse macrophage-like cell line RAW264.7 correlates with the degree of apoptosis and the generation of reactive oxygen species (ROS) [33,34]. However, the incorporation of 7.5% and 10% DPPE-PEG into liposomes reduced their uptake by ~30–45-fold and increased cell viability by ~20%–38%. Inclusion of DPPE-PEG also led to a decrease in ROS production [33], which diminished the toxic effects of liposomes [34]. Soenen et al. showed that adverse effects from cationic liposomes can be reduced by using ROS scavengers or a calcium channel blocker in 3T3 fibroblasts [87]. Based on these studies and many others [32,80,81,82,84,85,86,88], cytotoxicity could be readily detected in a wide range of cells upon increasing the zeta potential values of liposomes beyond ~30 mV, in a charge-dependent manner. In general, cationic liposomes are taken up by phagocytic cells to a greater extent and induce the formulation of ROS, which damages organelles (e.g., mitochondria), and promotes higher intracellular Na+ levels, compared to neutral and negatively charged liposomes [33,34,86,87,88,89,90].

Using animal models, Kedmi et al. showed that IV injection of cationic liposomes (+54.3 ± 6.1 mV) containing DOTAP induced hepatotoxicity and pro-inflammatory responses [80]. The cationic liposomes significantly reduced the body weight of C57BL/6 mice by up to 5.5% and increased liver enzymes in serum by 3–6-fold compared to neutral and negatively charged (−59.2 ± 4.9 mV) liposomes. Cationic liposomes also increased the amount of Th1 and Th17 cytokines by 10–24 fold at 2 h post-injection [80]. In rats, Knudsen and colleagues demonstrated that the administration of DOTAP/CHOL liposomes (+53 ± 2 mV) led to an upregulation of oxidative stress response gene (HMOX1) and the DNA repair enzyme, 8-oxoguanine glycosylase (OGG1), in the liver, as well as increased DNA strand breaks in the lungs [81]. While cationic liposomes are promising vehicles for gene delivery, understanding their toxicities to immune cells and regulation of inflammatory responses (discussed in Section 3) are critical for improving therapeutic benefits and avoiding immune toxicity.

### 2.2. Interactions with the Mononuclear Phagocyte System

While liposomes improve the pharmacokinetic profiles of therapeutics, they also redirect the therapeutic payloads to the MPS (previously known as the reticuloendothelial system, RES), [91,92,93]. The MPS is a collection of phagocytic cells (e.g., progenitor, monocytes and macrophages) located within the bone marrow, lymph nodes, spleen, and liver. The MPS is responsible for the removal of antigens from the body, and is the main site of liposome accumulation [94,95]. Several rodent studies have shown that Doxil accumulates within the liver and spleen (e.g., the two major organs of the MPS [91]) to a greater extent than other organs shortly after IV injections [96,97,98,99,100]. In cynomolgus monkeys, Kimelberg et al. found that liposomal encapsulation of [^3^H] methotrexate (MTX) caused a 160-fold increase in [3H]MTX uptake by the spleen compared to the free-form [^3^H]MTX [93]. It has been observed by many researchers that the redirection of liposomal chemotherapies to the MPS induces toxicity and depletion of immune cells, especially the Kupffer cells in the liver [31,101,102,103,104,105,106]. To study the impact of liposomes on the phagocytic function of MPS cells in mice, Allen et al. tested several compositions of egg PC-CHOL liposomes [101]. Injection (IV) of larger, multilamellar liposomes (synthesized using 200–450 nm filters) significantly decreased the phagocytic index of the liver by approximately 50% in mice, compared to using small unilamellar liposomes. The phagocytic index did not recover until 3 weeks post-injection, and further doses of liposomal injections led to accumulation in the spleen. Multiple injections caused a significant decrease in the phagocytic index of the spleen and a 2.5-fold increase in the spleen weight. The function and weight of the spleen did not recover until 8 days after the last liposome injection.

Several groups have also studied the impact of drug-containing liposomes on liver macrophages. Daemen et al. showed that IV injection of non-pegylated, short-circulating liposomal doxorubicin in rats reduced the number and phagocytic capacity of liver macrophages by up to 85–90%, compared to placebo liposomes [31]. The phagocytic capacity of liver macrophages was impaired for 8 days, and full recovery took 2 weeks. A follow-up study examined the effects of long-circulating liposomal doxorubicin containing ~4.8 mol% PE-PEG on the phagocytic activity of macrophages [106]. The pegylated liposomes reduced the phagocytic capacity and number of liver macrophages by 63% at 72 h after injection. Compared to non-pegylated liposomes, pegylated liposomes delayed the cytotoxic effects on liver macrophages by 24 h and modestly reduced the depletion of macrophage by 7%. While pegylation of liposomes reduces the blood protein adsorption and increases blood circulation time, the ultimate impact of pegylated and non-pegylated liposomes on macrophages and liver function was similar.

**Figure 2:**
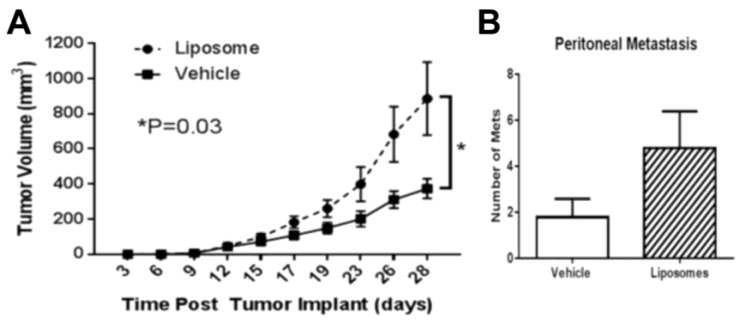
Intravenously injected liposomes impact cancer progression in vivo. Liposomal polarization of classically activated M1 tumor-associated macrophages (TAMs) to M2-like TAMs can result in carrier-induced immunosuppression and accelerated tumor development. Compared to vehicle control, empty (placebo) liposomes (**A**) accelerated tumor growth in a TC-1 subcutaneous mouse model and (**B**) increased the number of metastatic sites in an orthotopically implanted ID8-VEGF-GFP ovarian carcinoma mouse model on day 36. * P=0.03. (Cited from La-Beck et al., 2019 [107]).

Research groups have also shown that within the tumor microenvironment, liposomes can polarize tumor-associated macrophages (TAMs) [107,108,109]. The reprogramming of classically activated (M1) TAMs, which have a role in eradicating tumor cells in their early stages, to alternatively activated M2-like TAMs has been shown to promote tumor growth and metastasis, as shown in Figure 2 [110,111]. In an orthotopic ovarian carcinoma (ID8-VEGF-GFP) mouse model, La-Beck et al. showed that empty liposomes increased tumor volume by over 3-fold and doubled the number of metastatic sites, compared to the vehicle control [107]. Similarly, Sabnani et al. and Rajan et al. found that compared to the vehicle control (e.g., 5% dextrose solution, saline), empty pegylated liposomes accelerated the growth of subcutaneous TC-1 tumors in mice [109,112]. The accelerated tumor growth was attributed to diminished macrophage function and number of tumor-specific T cells [112], as well as a 66% decrease in M1-TAMs and a 100% increase in M2-TAMs [109]. Based on these results, strategies to overcome immunosuppression and tumor progression triggered by liposomes may potentially improve the efficacy of some liposomal anticancer drugs. New insights into the underlying molecular mechanisms may yield the key to designing new liposome-based immunotherapies.

### 2.3. Effect of Cholesterol Content on the Toxicity of Liposome

Cholesterol is commonly used to alter the mechanical properties and functionality of liposomes [113,114,115]. Cholesterol improves the stability and drug entrapment efficacy of liposomes by enabling more dense assembly of phospholipids [113,114,115,116,117,118]. Smaby et al. demonstrated that cholesterol condenses the lipids (e.g., from 63 to 44 Å^2^/molecule; 16:0–18:1 PC liposomes), thereby increasing the compressibility of liposomes from 123 to 401 mN/m [119]. The concentration of cholesterol can also impact the surface charge and permeability of liposomes [116]. Incorporation of 50 mol% cholesterol into 1,2-distearoyl-sn-glycero-3-phosphocholine (DSPC, 18:0 PC) liposomes reduced the zeta potential from 2 to −5.5 mV [120]. The presence of cholesterol also reduced the permeability coefficients of phospholipid vesicles to small molecules and ions [121]. In additional to structural stability and drug efflux related studies, several groups have examined the toxicity and immunogenicity of cholesterol. Adams et al. showed that injection of 5–10 mg of cholesterol into the mouse brain resulted in mild signs of edema and loss of internal neuronal structures [61]. Others have shown that free-form cholesterol induces macrophage apoptosis via the stiffening of the endoplasmic reticulum [122] and promotes the production of TNF-α and interleukin 6 (IL-6) [123]. Roerdink et al. showed that incorporation of 50 mol% of cholesterol into DSPC-based liposomes decreased their liver accumulation (from 70% to less than 40%) and extended the blood circulation half-life by 3-fold in rats [124]. It has also been shown that cholesterol-containing liposomes decrease macrophage uptake and immunoglobulin G (IgG) response in mice [125]. Similarly, incorporation of high levels (43 mol%) of cholesterol in ovalbumin-liposomes significantly reduced IgG response by 10-fold in mice [126]. In a Li 210 leukemia mouse model, Ganapathi et al. showed that cytarabine-loaded liposomes containing 50 mol% of cholesterol increased animal survival by up to ~20%, compared to using cytarabine-loaded liposomes containing 10 mol% of cholesterol or free-form cytarabine [127]. However, it is unclear if this enhanced cancer killing is due to increased cellular uptake of liposomes or an increase in the sustained release of cytarabine from cholesterol-containing liposomes. While the incorporation of cholesterol into liposomes mitigates macrophage uptake and antibody production, other studies have shown that cholesterol could activate the immune system by promoting interactions with C3, a protein associated with the complement system [128,129,130]. This is discussed extensively in Section 3.2.

## 3. Activation of the Immune System

Biomaterials introduced into the body can initiate immune responses in the host tissue. As shown in previous work, host defense mechanisms and inflammatory responses to biomaterials are known to be activated by the complement response [131]. The products from complement response activation may assist neutrophil aggregation and migration towards inflammatory sites [131]. Earlier research suggests that PEG-based hydrogels cause increased inflammatory responses compared to silicone-based hydrogels. This change was visualized through increased IL-1β expression in the host tissues after implantation of a PEG-based hydrogel [132]. It is also thought that complement activation can induce a foreign body reaction (FBR) [131]. During this process, host inflammatory cells and foreign body giant cells are activated to destroy the implanted material. Previous studies have demonstrated that thick macrophage capsules accumulate on PEG-based hydrogels as a result of the FBR when compared to other hydrogels lacking PEG [132].

Liposomes, like other nanoparticles used in drug delivery, interact with proteins in the body to trigger innate immune system responses [133,134,135]. Once liposomes have been administered to the patient, circulating proteins can adsorb to the surface of the liposome, thereby creating a protein corona unique to the characteristics of the liposome. The protein corona can then induce the activation or suppression of various immune responses [136]. Particularly, liposomes interact with complement proteins to activate the complement cascade and increase the body’s response to antigens [133,136,137]. The incorporation of PEG into liposomes has been shown to promote immunogenic responses via complement activation and the anti-PEG antibody production [138]. For these reasons, liposomes are being used as vaccine adjuvants to potentiate the immunological response to antigens. Antigen–liposomal complexes can increase and maintain exposure of the antigen in the lymph nodes, allowing for enhanced uptake by immature phagocytic antigen-presenting cells (e.g., macrophages and dendritic cells) [139,140,141]. A notable antigen-liposome adjuvant system, AS01, has been studied in clinical trials for multiple diseases [142], and was FDA approved as a shingles vaccine (Shingrix) in 2017.

The liposomal surface charge and size can impact the type and efficiency of the immune response. A study by Badiee et al. investigated the effects of the surface charge of recombinant rgp63-loaded liposomes on the immune response of mice with leishmania after subcutaneous injections. Neutral liposomes were found to promote a Th1 (e.g., IFN*γ*, IL-12) immune response more efficiently than positively charged liposomes. The largest IL-4, IgG2a, and IgG1 responses were induced by positively charged liposomes. A Th2 (e.g., IL-5, IL-6, IL-10) response, but not a Th1 immune response, was caused by liposomes with a negative surface charge [143]. In regards to liposome size, a follow-up study by Badiee et al. showed that 100 nm liposomes induce a Th2 response, while liposomes larger than 400 nm induce a Th1 response [144]. This was due, in part, to their altered pharmaceutics and intracellular trafficking. Similarly, Henriksen-Lacey et al. showed that intramuscularly injected ~200 nm CAF01 cationic liposomes induced the greatest IL-10 response in mice [145], while medium (500 nm) liposomes induced the largest IFN-γ and IL-1β response. The 200 nm and 1.5 μm liposomes were drained more rapidly by the lymph node, while the 500 nm liposomes were well retained at the site of injection [145].

### 3.1. Proinflammatory Cytokine Modulation

Liposomal interactions with blood cells lead to cytokine production and the activation of the immune system [75,80,146,147]. The activation of the complement cascade also promotes the secretion of cytokines from immune cells [148,149]. This concept is well demonstrated in the first liposomal gene silencing clinical trial (NCT00882180). Liposomes were loaded with siRNAs that were chemically modified to minimize immunostimulation and administered intravenously. However, there was still a dose-dependent induction of proinflammatory cytokines. Cytokines, including IL-6, IP-10, granulocyte colony-stimulating factor, and TNF-α, peaked 6 h post-injection. One of the largest liposome doses, 1.25 mg/kg, resulted in the most IL-6 present in the patients’ blood (~9000 pg/mL). There was also a ~3–10 fold increase in Bb complement protein for patients who received a dose larger than 0.2 mg/kg [75]. Yamamoto et al. demonstrated that cytokine induction by HEPC-based liposomes is comparable to that of a potent activator of monocytes and macrophages, lipopolysaccharides (LPS). Also, incubation of liposomes with human peripheral blood induced monocyte-derived cytokines (IL-6, IL-10, IL-1β, IFN-y and TNF-a) but not lymphocyte-derived cytokines (IL-2, IL-4 and IL-5). In the same study, it was shown that the size of liposomes directly affects the degree of cytokine release, in vitro, in a size-dependent manner. Liposomes with an 800 nm diameter induced the production of 6000 pg/mL of IL-1β, while 50 nm liposomes produced virtually none [146].

For gene delivery using liposome–DNA complexes, previous reports have shown that nonspecific cytokine production from toll-like receptor-9 (TLR9) positive immune cells occurs due to unmethylated CpG dinucleotides (CpG motifs) abundantly present in pDNA [150,151]. Liposome–DNA complexes also induced the production of type I interferons (IFNs), irrespective of the frequency of CpG motifs in DNA and the expression of TLR9 [152]. However, a study conducted by Yasuda et al. showed that immune activation by liposome–DNA complexes is highly dependent on the type of cationic liposome. In the murine macrophage-like cell line, RAW264.7, non-CpG containing Lipofectamine2000 liposomes (+35.1 mV) induced the largest cytokine production (500–900 pg/mL), while DOTMA/DOPE and DOTMA/CHOL liposomes (+32.7 mV) produced less than one-fifth of that amount [153]. Furthermore, Lipofectamine2000 liposomes containing non-CpG motif DNA induced IFN-β and IL-6 production by macrophages cultured from TLR9 deficient mice [153]. These liposome-induced inflammatory responses are critical to consider for clinical applications, as increased levels of cytokines can cause side effects (e.g., grade 1–2 chills/rigors, flu-like symptoms) [75,154].

### 3.2. Recognition of Liposomes via Complement Activation

**Figure 3:**
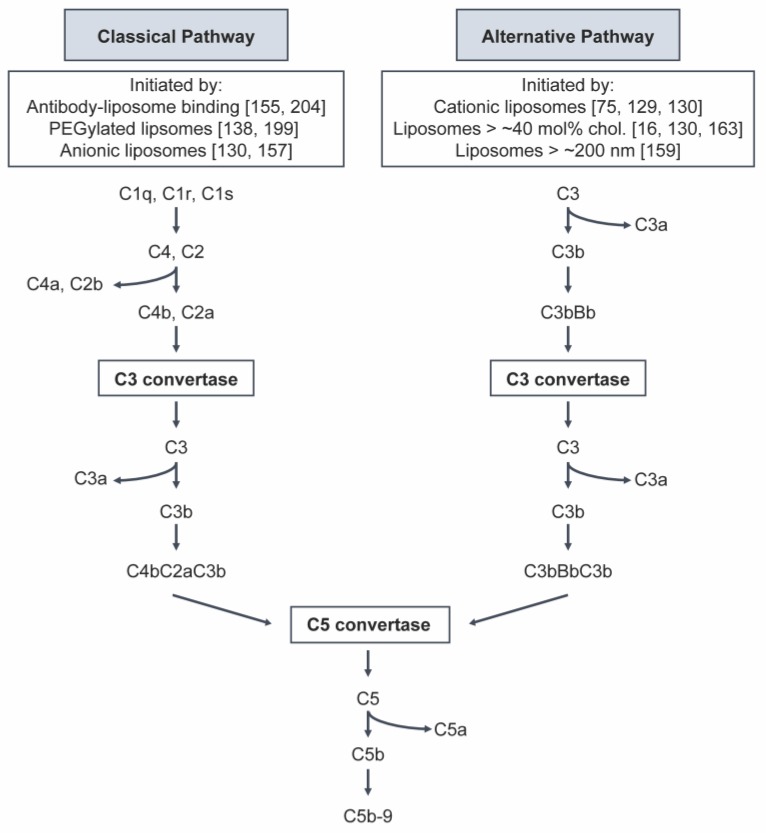
Schematic overview of the complement system highlighting two of the main activation pathways (classical vs. alternative). Previous studies have shown that classical pathway activation is initiated by antibody-liposome binding, pegylated liposomes, and anionic liposomes. The alternative pathway can be initiated by cationic liposomes, liposomes containing more than 40 mol% cholesterol, and liposomes larger than 200 nm in diameter. During the complement cascade, C3b opsonin covalently binds to the surface of the liposome, marking it for removal by the MPS. The released anaphylatoxins (C3a, C4a, and C5a) prompt the activation of leukocytes.

The complement system is a part of the innate immune system responsible for the recognition of foreign objects in the body [148]. It is composed of over 30 plasma proteins that interact with each other to opsonize (e.g., C3b) and clear liposomes, as well as induce a series of inflammatory responses by liberating anaphylatoxins (e.g., C3a, C4a, C5a) (Figure 3) [137,148]. Early reports indicated that liposomes activate the alternative pathway via C3 binding and conversion, which is independent of antibody recognition. Cunningham et al. showed that C3 conversion occurs when cationic liposomes are incubated in serum from C1r-deficient and C2-deficient patients [129]. Liposomes without cationic lipids or with less than 20 mol% of cholesterol were unable to convert C3 in C2-deficient serum [129]. However, later reports show that complement activation is dependent on the surface chemistry and composition of liposomes [155,156,157]. A study by Chonn and colleagues showed that liposomes containing 20 mol% of anionic lipids activated the classical pathway in a Ca(2+)-dependent manner. Calcium is required in the classical pathway, as it is used for C1qr2s2 complex formation. Cationic liposomes activated the alternative pathway by reducing the C3-C9 levels in Ca(2+)-depleted serum. In addition, liposomes containing unsaturated lipids or 45 mol% of cholesterol promoted complement protein interactions. In contrast, neutral liposomes, even at a high lipid concentration of 50 mM, did not activate the complement pathways [130]. Bradley et al. showed that anionic liposomes can also activate the classical pathway in an antibody-independent manner via direct binding of C1q to the liposomal surface [157]. Liposome size, pegylation, and cholesterol content can also contribute to the degree and nature of complement activation. For example, studies have suggested that larger liposomes, over 200 nm in diameter, were associated with an increase in complement protein opsonization and alternative pathway activation [158,159]. Liposomes containing PEG typically induce IgM antibody production (see Section 3.3), followed by (classical) complement activation [138]. A study by Alving et al. showed a cholesterol-dependent activation, with liposomes containing 73 mol% cholesterol promoting the greatest classical pathway activation [160]. While examining the hypersensitivity reactions (HSR) induced by different components of Doxil, Szebeni et al. demonstrated that negatively charged PEG-PE is a potent C protein activator, and the degree of complement activation correlates with HSR [161]. When PEG-PE-based liposomes were incubated with human serum, there was a 2.5–4-fold increase in soluble complement S5b-9 (C5b-9), compared to HSPC-based liposomes which had no impact on complement activation [161]. Activation of C5b-9 following exposure to pegylated liposomes has been associated with development of HSR [162], which is discussed in the following section.

### 3.3. Hypersensitivity Reactions

Liposomes have also been shown to induce HSR [163,164,165,166,167,168,169,170]. HSR are non-IgE-mediated pseudo-allergic immune responses, which usually develop immediately after IV infusion when the body is exposed to an antigen or liposomes [162]. While 6.8% of the 705 patients who received Doxil for refractory AIDS-related Kaposi’s sarcoma experienced HSR with symptoms ranging from dyspnea and tachypnea to hypotension and hypertension, the underlying causes remain unclear [170,171], with some researchers attributing similar reactions to complement activation. [172,173]. Later reports have shown that HSR are correlated with PEG-induced complement activation, however high levels of C activation may be necessary, suggesting the involvement of other pathogenic factors [161,162,174]. This subset of HSR, known as *C* activation-related pseudoallergy (CARPA), can be reduced by slowing the rate of infusion, diluting the Doxil dose, or premedicating with a corticosteroid [175]. In a 4-patient study, grade 3 HSR induced by Doxil occurred almost immediately after the start of infusion in all patients and treatment was stopped. Premedication with ranitidine and hydroxyzine prior to the resumption of Doxil infusion eliminated HSR in 3 of the 4 patients [166]. Similarly, data analysis performed by Chanan-Khan et al. showed that, on average, 8% of people who received Doxil experienced HSR [162]. Additionally, 3% of refractory ovarian cancer patients who were pretreated with corticosteroids and antihistamines to minimize adverse reactions still experienced HSR. Chanan-Khan et al. further showed that HSR occurrence can be as high as 45% for patients receiving Doxil. Within this Phase 1 clinical study, 92% of the patients who experienced HSR had significantly elevated Plasma SC5b-9 levels [162]. HSR have been suggested to be caused by the liposomal vehicle of Doxil rather than the encapsulated drug, as these reactions are not known to occur with standard doxorubicin [161,162]. It should also be noted that other chemotherapies, such as Taxol (paclitaxel), which relies on a formulation vehicle, and carboplatin, are known to cause HSR [176,177].

Using pig models, Szebeni et al. showed that pulmonary hypertension reactions are dependent on the composition, size, and administration method of liposomes [165]. Large, neutral, multilamellar liposomes composed of 1,2-dimyristoyl-*sn*-glycero-3-phosphocholine (DMPC, 14:0 PC) and DSPC induced pulmonary hypertension, while smaller liposomes (<200 nm) caused no hemodynamic changes [165]. An additional study by Szebeni et al. showed that highly negatively charged liposomes containing L-α-phosphatidylglycerol (soy PG) initiated CARPA to the greatest extent in pigs, suggesting that surface charge may also play a role [178]. Fülöp et al. showed a 300%–600% increase in pulmonary arterial blood pressure after the infusion of pegylated liposomal prednisolone sodium phosphate in pigs [164]. The risk of these side effects was reduced by using a slow infusion protocol (0.04 mL/kg/h) with a 3-step dose escalation [164]. It is now recommended that Doxil be administered at 1 mg/min to help minimize these adverse reactions [179]. Within a 2018 Nature Nanotechnology Perspective, authors suggested that the next steps for minimizing HSR and CARPA are identifying induced biomarkers and understanding the underlying mechanisms via in vitro assays [180].

### 3.4. Anti-PEG Response

**Figure 4:**
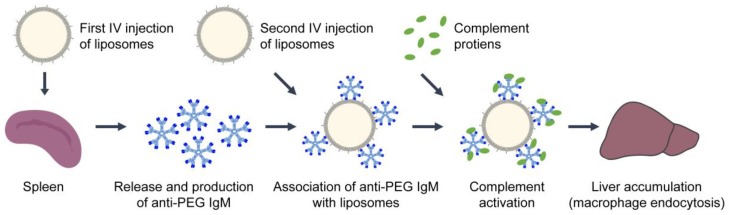
Schematic overview of the events that alter the pharmacokinetics of repeated intravenous (IV) doses of pegylated liposomes. The first dose of pegylated liposomes stimulates the production of anti-PEG IgM antibodies by B cells within the spleen. This leads to the accelerated blood clearance of subsequent doses, during which circulating anti-PEG IgMs bind to the liposomes, initiating classical complement activation and decreasing blood circulation half-life.

Liposomal interactions with immune cells can cause antibody production against liposome components, such as PEG [36,138,181,182,183,184,185]. The rapid production of antibodies (e.g., IgM, IgE, IgG) from B lymphocytes, in a T cell-independent manner, is another mechanism in the recognition and removal of liposomes from the body [186]. Antibody binding impacts the blood circulation time and treatment efficacy of liposomes, and initiates classical complement activation. IgM antibodies are the first produced, by the spleen, in response to an antigen or liposomes, as seen in Figure 4 [183,187,188]. One of the first studies to notice an increase in antibody production was by Bucke and colleagues in 1998 when studying the biodistribution of liposomes with different surface modifications [189]. Additionally, Cheng et al. were one of the first to show that anti-PEG IgM monoclonal antibodies were generated by the spleen of mice in response to PEG-immunoconjugates [190]. Based on blood concentration levels and binding, IgM antibodies were more efficient in clearing the PEG-immunoconjugates from the blood than IgG [190].

Since then, several groups have studied antibody production in preclinical animal models, which helped to explain the accelerated blood clearance seen after repeated dosing of liposomes. Ishida et al. showed that pegylated liposomes incubated with serum derived from rats pre-injected with liposomes had an increased liposome-protein binding index by approximately 2-fold compared to serum obtained from rats that were not pre-injected with liposomes [36]. Also, there was a 17-fold increase in IgM binding to pegylated liposomes compared to the non-pegylated liposomes. A follow-up study by Ishida and colleagues revealed a T cell-independent response to pegylated-liposomes in both Wistar rats and BALB/c nu/nu mice [181]. A relatively low anti-PEG IgG response was seen 3–5 days after injection of pegylated liposomes and a potent IgM response occurred 3–10 days post injection in Wistar rats. In the absence of T-cells, a ~9-fold increase in IgM was observed 10 days post-injection in BALB/c nu/nu mice [181]. Additionally, using a pig model, Kozma et al. showed that anti-PEG IgM concentration in blood peaks at 7–9 days after low dose IV injection of pegylated liposomes [174]. The anti-PEG IgM levels remained elevated above normal, by approximately 10-fold, for 6 weeks. Using ex vivo spleen cells derived from pigs pre-injected with pegylated liposomes, they showed an increase in IgM+ B-cells bound to Doxil from 1.88% to 5.50% [174]. The association between the underlying mechanism of PEG immunogenicity and the “accelerated blood clearance” of subsequent liposome injections will be discussed in the next section. In contrast to the immunogenic effects, it is critical to point out that PEG may have additional immunosuppressive effects by decreasing inflammation and fibrosis, as seen in organ preservation [191], which warrants further investigation.

### 3.5. Accelerated Blood Clearance

As previously mentioned, the addition of PEG to liposomes can increase their blood circulation half-life and prolong the uptake by the MPS [48]. It is also well accepted that after repeated injections of pegylated liposomes, they lose their “stealth” ability and are more quickly cleared from the blood [138,192,193,194,195,196,197,198,199,200,201,202,203,204]. This phenomenon, known as “accelerated blood clearance” (ABC), is caused by the abundant secretion of anti-PEG IgM, IgE, and IgG, followed by the opsonization of C protein fragments (complement activation), and finally uptake by macrophages, depicted in Figure 4 [138,192,193,194,195,196,197,198,199,200,201,202,203]. Dams et al. first observed a dramatic change in the circulatory half-life of intravenously injected 99mTc-labeled pegylated-liposomes in rats and monkeys, but not mice [192]. In a male rhesus monkey, the blood circulation half-life of the second dose of pegylated-liposomes dropped from 87.5 h to 14.2 h. Although further liposome injections after the second dose had similar kinetics as the first, the ABC of the second dose occurred if it was administered 1–4 weeks after the first. Rats transfused with blood or serum from rats that were previously injected with liposomes also experienced ABC. The percent of the injected dose of liposomes in blood transfused rats at 4 h dropped from 52% to less than 23% in the blood, suggesting that the ABC is caused by a soluble serum factor [192].

Studying the ABC of liposomal chemotherapy in rodents may not be ideal, as Laverman et al. confirmed that Doxil injections in rats lead to hepatosplenic macrophage depletion, and therefore a second injection of Doxil did not experience ABC [193]. However, if the first dose was empty pegylated liposomes, an injection of Doxil within one week was cleared rapidly from circulation in rats [193]. Similarly, in Beagle dogs, Suzuki et al. showed that repeated doses of Doxil at 20 mg/m^2^ did not have altered pharmaceutics, but repeated doses less than 2 mg/m^2^ were more rapidly cleared from the blood and elicited an IgM response [201]. The half-life of the second injection at 2 mg/m^2^ was reduced from 24.1 h to 1.5 h. Larger doses of Doxil could have caused damage to B cells within the spleen, attenuating antibody production, or the uptake capacity of Kupffer cells may have been saturated/suppressed by the first large dose [201]. It is critical to mention that the clinical relevance of ABC phenomenon induced by repeated injection of pegylated liposomes remains debatable, since Gabizon et al. observed a significant decrease in clearance (*P* < 0.0001) from the 1st through the 3rd cycle of pegylated liposomal doxorubicin in humans [205]. The wide use of PEG in healthcare, hygiene, and beauty products suggests that most patients will likely have pre-existing anti-PEG antibodies [206], which could potentially impact the degree of ABC in patients. Other parameters that have been shown to impact the ABC of liposomes are lipid dose, with increasing amounts of the prior dose altering the pharmacokinetics of the subsequent injections in a sigmoid manner [196], liposome composition (with unsaturated lipids causing a more pronounced ABC [195]), and the time and frequency of injections [192,194,195,207].

## 4. Conclusions

For two and a half decades, liposomal drug formulations have been administered in the clinic for the treatment of a variety of ailments, ranging from cancer to fungal disease. They boast an array of advantageous features, including biocompatibility, tunable properties, and capacity for loading hydrophilic and hydrophobic agents, making them convenient drug delivery vehicles. However, despite their clinical relevance and therapeutic potential, there is still a scarcity of knowledge regarding the downsides associated with the administration of liposomes. This mini-review presents a summary of existing knowledge regarding such limitations, divided into two main sections: (1) Toxicological Evaluation of Liposomes and their Building Blocks, and (2) Activation of the Immune System. One of the main toxicological concerns is that cationic liposomes, which are primarily used for nucleic acid delivery, can be toxic to macrophages and reduce their secretion of important immunomodulators. Additionally, following IV injection, liposomes end up sequestered in the organs of the MPS, such as the spleen and liver. This induces toxicity in these organs, causing the depletion of cells that are critical for proper immune system function. As for activation of the immune system, liposomes can induce an inflammatory response that is characterized by the release of pro-inflammatory, monocyte-derived cytokines. Similarly, liposomes have been shown to activate the complement pathway, though the degree of activation is dependent on several factors such as size, charge, and mol% of cholesterol. Another important mechanism of immune activation is the anti-PEG response, which occurs when pegylated liposomal formulations activate the production of PEG antibodies by B lymphocytes. This leads to recognition and clearance by the immune system. In fact, repeated administrations of pegylated liposomes have been shown to cause accelerated blood clearance, robbing liposomes of their stealth capabilities. This underscores the idea that the dosing of liposomal agents must be carefully optimized to avoid premature clearance and maximize the therapeutic benefits of each injection. Depending on injection speed, liposomes can also induce hypersensitivity reactions, including cardiopulmonary distress. Mode of delivery and speed of administration are therefore key considerations for clinical applications of liposomal formulations. Overall, liposome-mediated toxicity to healthy tissues, coupled with the activation of the immune system, raise important concerns regarding their use. While liposomes are an attractive tool to reduce the dose-limiting side effects of therapeutics in patients, the liposomes themselves can impart new toxicities that are still not fully understood. From our perspective, a neutral liposomal formulation consisting of a relatively low mol% of cholesterol and PEG (in order to balance stability and high blood circulation half-life with low toxicity and immune system recognition) should be considered for chemotherapy delivery. With regards to gene delivery, a liposomal surface charge less than 30 mV should be considered to minimize toxicities. There is still much work to be done to understand the mechanisms through which liposomes interact with the immune system before the full potential of liposome-based systems for gene and drug delivery can be achieved.

## References

[1] Torchilin V.P. (2005). Recent advances with liposomes as pharmaceutical carriers. Nat. Rev. Drug Discov..

[2] Akbarzadeh A., Rezaei-Sadabady R., Davaran S., Joo S.W., Zarghami N., Hanifehpour Y., Samiei M., Kouhi M., Nejati-Koshki K. (2013). Liposome: Classification, preparation, and applications. Nanoscale Res. Lett..

[3] Bozzuto G., Molinari A. (2015). Liposomes as nanomedical devices. Int. J. Nanomed..

[4] Szoka F., Papahadjopoulos D. (1978). Procedure for preparation of liposomes with large internal aqueous space and high capture by reverse-phase evaporation. Proc. Natl. Acad. Sci. USA.

[5] Bangham A.D., Horne R.W. (1964). Negative staining of phospholipids and their structural MODIFICATION BY surface-active agents as observed in the electron microscope. J. Mol. Biol..

[6] Bangham A.D., Standish M.M., Watkins J.C. (1965). Diffusion of univalent ions across the lamellae of swollen phospholipids. J. Mol. Biol..

[7] Barenholz Y. (2012). Doxil®—The first FDA-approved nano-drug: Lessons learned. J. Control Release.

[8] Bulbake U., Doppalapudi S., Kommineni N., Khan W. (2017). Liposomal Formulations in Clinical Use: An Updated Review. Pharmaceutics.

[9] Huang H.C., Mallidi S., Obaid G., Sears B., Tangutoori S., Hasan T., Hamblin M.R., Avci P. (2015). 23-Advancing photodynamic therapy with biochemically tuned liposomal nanotechnologies. Applications of Nanoscience in Photomedicine.

[10] Chrai S.S., Murari R., Ahmad I. (2001). Liposomes (a review)-Part one: Manufacturing issues. Biopharm.-Appl. Technol. Biopharm. Dev..

[11] Dua J., Rana A., Bhandari A. (2012). Liposome: Methods of preparation and applications. Int. J. Pharm. Stud. Res..

[12] Powers D., Nosoudi N. (2019). Liposomes; from synthesis to targeting macrophages. Biomed. Res..

[13] Gubernator J. (2011). Active methods of drug loading into liposomes: Recent strategies for stable drug entrapment and increased in vivo activity. Expert Opin. Drug Deliv..

[14] Sur S., Fries A.C., Kinzler K.W., Zhou S., Vogelstein B. (2014). Remote loading of preencapsulated drugs into stealth liposomes. Proc. Natl. Acad. Sci. USA.

[15] Yu L.X., Li B.V. (2014). FDA Bioequivalence Standards.

[16] Fahr A., Hoogevest P.V., May S., Bergstrand N., Leigh M.L.S. (2005). Transfer of lipophilic drugs between liposomal membranes and biological interfaces: Consequences for drug delivery. Eur. J. Pharm. Sci..

[17] Bhakay A., Rahman M., Dave R.N., Bilgili E. (2018). Bioavailability Enhancement of Poorly Water-Soluble Drugs via Nanocomposites: Formulation(-)Processing Aspects and Challenges. Pharmaceutics.

[18] Bressler N.M. (2001). Photodynamic therapy of subfoveal choroidal neovascularization in age-related macular degeneration with verteporfin: Two-year results of 2 randomized clinical trials-tap report 2. Arch. Ophthalmol..

[19] Schmidt-Erfurth U., Hasan T., Gragoudas E., Michaud N., Flotte T.J., Birngruber R. (1994). Vascular targeting in photodynamic occlusion of subretinal vessels. Ophthalmology.

[20] Richter A.M., Cerruti-Sola S., Sternberg E.D., Dolphin D., Levy J.G. (1990). Biodistribution of tritiated benzoporphyrin derivative (3H-BPD-MA), a new potent photosensitizer, in normal and tumor-bearing mice. J. Photochem. Photobiol. B Biol..

[21] Sercombe L., Veerati T., Moheimani F., Wu S.Y., Sood A.K., Hua S. (2015). Advances and Challenges of Liposome Assisted Drug Delivery. Front. Pharmacol..

[22] Vieira D.B., Gamarra L.F. (2016). Getting into the brain: Liposome-based strategies for effective drug delivery across the blood-brain barrier. Int. J. Nanomed..

[23] Drummond D.C., Meyer O., Hong K., Kirpotin D.B., Papahadjopoulos D. (1999). Optimizing liposomes for delivery of chemotherapeutic agents to solid tumors. Pharm. Rev..

[24] Olusanya T.O.B., Haj Ahmad R.R., Ibegbu D.M., Smith J.R., Elkordy A.A. (2018). Liposomal Drug Delivery Systems and Anticancer Drugs. Molecules.

[25] Chow T.H., Lin Y.Y., Hwang J.J., Wang H.E., Tseng Y.L., Wang S.J., Liu R.S., Lin W.J., Yang C.S., Ting G. (2009). Improvement of biodistribution and therapeutic index via increase of polyethylene glycol on drug-carrying liposomes in an HT-29/luc xenografted mouse model. Anticancer Res..

[26] Rahman A.M., Yusuf S.W., Ewer M.S. (2007). Anthracycline-induced cardiotoxicity and the cardiac-sparing effect of liposomal formulation. Int. J. Nanomed..

[27] Xing M., Yan F., Yu S., Shen P. (2015). Efficacy and Cardiotoxicity of Liposomal Doxorubicin-Based Chemotherapy in Advanced Breast Cancer: A Meta-Analysis of Ten Randomized Controlled Trials. PLoS ONE.

[28] Kanter P.M., Bullard G.A., Pilkiewicz F.G., Mayer L.D., Cullis P.R., Pavelic Z.P. (1993). Preclinical toxicology study of liposome encapsulated doxorubicin (TLC D-99): Comparison with doxorubicin and empty liposomes in mice and dogs. In Vivo.

[29] Drummond D.C., Noble C.O., Guo Z., Hong K., Park J.W., Kirpotin D.B. (2006). Development of a Highly Active Nanoliposomal Irinotecan Using a Novel Intraliposomal Stabilization Strategy. Cancer Res..

[30] Wilhelm S., Tavares A.J., Dai Q., Ohta S., Audet J., Dvorak H.F., Chan W.C.W. (2016). Analysis of nanoparticle delivery to tumours. Nat. Rev. Mater..

[31] Daemen T., Hofstede G., Ten Kate M.T., Bakker-Woudenberg I.A.J.M., Scherphof G.L. (1995). Liposomal doxorubicin-induced toxicity: Depletion and impairment of phagocytic activity of liver macrophages. Int. J. Cancer.

[32] Filion M.C., Phillips N.C. (1997). Toxicity and immunomodulatory activity of liposomal vectors formulated with cationic lipids toward immune effector cells. Biochim. Biophys. Acta.

[33] Takano S., Aramaki Y., Tsuchiya S. (2003). Physicochemical properties of liposomes affecting apoptosis induced by cationic liposomes in macrophages. Pharm. Res..

[34] Aramaki Y., Takano S., Tsuchiya S. (1999). Induction of apoptosis in macrophages by cationic liposomes. FEBS Lett..

[35] Watson D.S., Endsley A.N., Huang L. (2012). Design considerations for liposomal vaccines: Influence of formulation parameters on antibody and cell-mediated immune responses to liposome associated antigens. Vaccine.

[36] Ishida T., Ichihara M., Wang X., Yamamoto K., Kimura J., Majima E., Kiwada H. (2006). Injection of PEGylated liposomes in rats elicits PEG-specific IgM, which is responsible for rapid elimination of a second dose of PEGylated liposomes. J. Control Release.

[37] Landesman-Milo D., Peer D. (2012). Altering the immune response with lipid-based nanoparticles. J. Control Release.

[38] Li J., Wang X., Zhang T., Wang C., Huang Z., Luo X., Deng Y. (2015). A review on phospholipids and their main applications in drug delivery systems. Asian J. Pharm. Sci..

[39] Miranda D., Lovell J.F. (2016). Mechanisms of light-induced liposome permeabilization. Bioeng. Transl. Med..

[40] Luo D., Li N., Carter K.A., Lin C., Geng J., Shao S., Huang W.-C., Qin Y., Atilla-Gokcumen G.E., Lovell J.F. (2016). Rapid Light-Triggered Drug Release in Liposomes Containing Small Amounts of Unsaturated and Porphyrin-Phospholipids. Small (Weinheim an der Bergstrasse Germany).

[41] Kohli A.G., Kierstead P.H., Venditto V.J., Walsh C.L., Szoka F.C. (2014). Designer lipids for drug delivery: From heads to tails. J. Control Release Off. J. Control. Release Soc..

[42] Tsui F.C., Ojcius D.M., Hubbell W.L. (1986). The intrinsic pKa values for phosphatidylserine and phosphatidylethanolamine in phosphatidylcholine host bilayers. Biophys J..

[43] Inglut C.T., Baglo Y., Liang B.J., Cheema Y., Stabile J., Woodworth G.F., Huang H.C. (2019). Systematic Evaluation of Light-Activatable Biohybrids for Anti-Glioma Photodynamic Therapy. J. Clin. Med..

[44] Lovell J.F., Jin C.S., Huynh E., Jin H., Kim C., Rubinstein J.L., Chan W.C.W., Cao W., Wang L.V., Zheng G. (2011). Porphysome nanovesicles generated by porphyrin bilayers for use as multimodal biophotonic contrast agents. Nat. Mater..

[45] Irby D., Du C., Li F. (2017). Lipid-Drug Conjugate for Enhancing Drug Delivery. Mol. Pharm..

[46] Signorell R.D., Luciani P., Brambilla D., Leroux J.C. (2018). Pharmacokinetics of lipid-drug conjugates loaded into liposomes. Eur. J. Pharm. Biopharm..

[47] Baglo Y., Liang B.J., Robey R.W., Ambudkar S.V., Gottesman M.M., Huang H.-C. (2019). Porphyrin-lipid assemblies and nanovesicles overcome ABC transporter-mediated photodynamic therapy resistance in cancer cells. Cancer Lett..

[48] Immordino M.L., Dosio F., Cattel L. (2006). Stealth liposomes: Review of the basic science, rationale, and clinical applications, existing and potential. Int. J. Nanomed..

[49] Nunes S.S., Fernandes R.S., Cavalcante C.H., da Costa César I., Leite E.A., Lopes S.C.A., Ferretti A., Rubello D., Townsend D.M., de Oliveira M.C. (2019). Influence of PEG coating on the biodistribution and tumor accumulation of pH-sensitive liposomes. Drug Deliv. Transl. Res..

[50] Swenson C.E., Bolcsak L.E., Batist G., Guthrie T.H.J., Tkaczuk K.H., Boxenbaum H., Welles L., Chow S.-C., Bhamra R., Chaikin P. (2003). Pharmacokinetics of doxorubicin administered i.v. as Myocet (TLC D-99; liposome-encapsulated doxorubicin citrate) compared with conventional doxorubicin when given in combination with cyclophosphamide in patients with metastatic breast cancer. Anti-Cancer Drugs.

[51] Hamilton A., Biganzoli L., Coleman R., Mauriac L., Hennebert P., Awada A., Nooij M., Beex L., Piccart M., Van Hoorebeeck I. (2002). EORTC 10968: A phase I clinical and pharmacokinetic study of polyethylene glycol liposomal doxorubicin (Caelyx®, Doxil) at a 6-week interval in patients with metastatic breast cancer. Ann. Oncol..

[52] Corbo C., Molinaro R., Taraballi F., Toledano Furman N.E., Sherman M.B., Parodi A., Salvatore F., Tasciotti E. (2016). Effects of the protein corona on liposome-liposome and liposome-cell interactions. Int. J. Nanomed..

[53] Corbo C., Molinaro R., Parodi A., Toledano Furman N.E., Salvatore F., Tasciotti E. (2016). The impact of nanoparticle protein corona on cytotoxicity, immunotoxicity and target drug delivery. Nanomed. (Lond.).

[54] Barbero F., Russo L., Vitali M., Piella J., Salvo I., Borrajo M.L., Busquets-Fité M., Grandori R., Bastús N.G., Casals E. (2017). Formation of the Protein Corona: The Interface between Nanoparticles and the Immune System. Semin. Immunol..

[55] Caracciolo G. (2015). Liposome-protein corona in a physiological environment: Challenges and opportunities for targeted delivery of nanomedicines. Nanomedicine.

[56] Hadjidemetriou M., Al-Ahmady Z., Mazza M., Collins R.F., Dawson K., Kostarelos K. (2015). In Vivo Biomolecule Corona around Blood-Circulating, Clinically Used and Antibody-Targeted Lipid Bilayer Nanoscale Vesicles. ACS Nano.

[57] Gessner A., Lieske A., Paulke B.-R., Müller R.H. (2003). Functional groups on polystyrene model nanoparticles: Influence on protein adsorption. J. Biomed. Mater. Res. Part A.

[58] Gessner A., Waicz R., Lieske A., Paulke B.R., Mäder K., Müller R.H. (2000). Nanoparticles with decreasing surface hydrophobicities: Influence on plasma protein adsorption. Int. J. Pharm..

[59] Papahadjopoulos D., Poste G., Schaeffer B.E. (1973). Fusion of mammalian cells by unilamellar lipid vesicles: Influence of lipid surface charge, fluidity and cholesterol. Biochim. Biophys. Acta (BBA)-Biomembr..

[60] Pagano R.E., Huang L., Wey C. (1974). Interaction of phospholipid vesicles with cultured mammalian cells. Nature.

[61] Adams D.H., Joyce G., Richardson V.J., Ryman B.E., Wisniewski H.M. (1977). Liposome toxicity in the mouse central nervous system. J. Neurol. Sci..

[62] Shi M., Anantha M., Wehbe M., Bally M.B., Fortin D., Roy L.-O., Charest G., Richer M., Paquette B., Sanche L. (2018). Liposomal formulations of carboplatin injected by convection-enhanced delivery increases the median survival time of F98 glioma bearing rats. J. Nanobiotechnol..

[63] Fan Y., Sahdev P., Ochyl L.J., Akerberg J., Moon J.J. (2015). Cationic liposome-hyaluronic acid hybrid nanoparticles for intranasal vaccination with subunit antigens. J. Control Release.

[64] Hernandez-Caselles T., Villalain J., Gomez-Fernandez J.C. (1993). Influence of liposome charge and composition on their interaction with human blood serum proteins. Mol. Cell. Biochem..

[65] Heath T.D., Lopez N.G., Papahadjopoulos D. (1985). The effects of liposome size and surface charge on liposome-mediated delivery of methotrexate-gamma-aspartate to cells in vitro. Biochim. Biophys. Acta.

[66] Parnham M.J., Wetzig H. (1993). Toxicity screening of liposomes. Chem. Phys. Lipids.

[67] Lappalainen K., Jääskeläinen I., Syrjänen K., Urtti A., Syrjänen S. (1994). Comparison of Cell Proliferation and Toxicity Assays Using Two Cationic Liposomes. Pharm. Res..

[68] Lentz B.R. (2003). Exposure of platelet membrane phosphatidylserine regulates blood coagulation. Prog. Lipid Res..

[69] Zbinden G., Wunderli-Allenspach H., Grimm L. (1989). Assessment of thrombogenic potential of liposomes. Toxicology.

[70] Ishiwata H., Suzuki N., Ando S., Kikuchi H., Kitagawa T. (2000). Characteristics and biodistribution of cationic liposomes and their DNA complexes. J. Control Release.

[71] Office of Medical Products and Tobacco, Center for Drug Evaluation and Research (2018). Liposome Drug Products: Chemistry, Manufacturing, and Controls; Human Pharmacokinetics and Bioavailability; and Labeling Documentation.

[72] White R.E., Evans D.C., Hop C.E., Moore D.J., Prakash C., Surapaneni S., Tse F.L. (2013). Radiolabeled mass-balance excretion and metabolism studies in laboratory animals: A commentary on why they are still necessary. Xenobiotica.

[73] Simoes S., Filipe A., Faneca H., Mano M., Penacho N., Duzgunes N., de Lima M.P. (2005). Cationic liposomes for gene delivery. Expert Opin. Drug Deliv..

[74] Balazs D.A., Godbey W. (2011). Liposomes for use in gene delivery. J. Drug Deliv..

[75] Tabernero J., Shapiro G.I., LoRusso P.M., Cervantes A., Schwartz G.K., Weiss G.J., Paz-Ares L., Cho D.C., Infante J.R., Alsina M. (2013). First-in-Humans Trial of an RNA Interference Therapeutic Targeting VEGF and KSP in Cancer Patients with Liver Involvement. Cancer Discov..

[76] Zhou J., Shum K.-T., Burnett J.C., Rossi J.J. (2013). Nanoparticle-Based Delivery of RNAi Therapeutics: Progress and Challenges. Pharmaceuticals (Basel).

[77] Akhtar S., Benter I.F. (2007). Nonviral delivery of synthetic siRNAs in vivo. J. Clin. Investig..

[78] Li W., Szoka F.C. (2007). Lipid-based Nanoparticles for Nucleic Acid Delivery. Pharm. Res..

[79] Lv H., Zhang S., Wang B., Cui S., Yan J. (2006). Toxicity of cationic lipids and cationic polymers in gene delivery. J. Control Release.

[80] Kedmi R., Ben-Arie N., Peer D. (2010). The systemic toxicity of positively charged lipid nanoparticles and the role of Toll-like receptor 4 in immune activation. Biomaterials.

[81] Knudsen K.B., Northeved H., Kumar P.E., Permin A., Gjetting T., Andresen T.L., Larsen S., Wegener K.M., Lykkesfeldt J., Jantzen K. (2015). In vivo toxicity of cationic micelles and liposomes. Nanomedicine.

[82] Hattori Y., Shimizu S., Ozaki K.I., Onishi H. (2019). Effect of Cationic Lipid Type in Folate-PEG-Modified Cationic Liposomes on Folate Receptor-Mediated siRNA Transfection in Tumor Cells. Pharmaceutics.

[83] Zelphati O., Szoka F.C. (1996). Intracellular Distribution and Mechanism of Delivery of Oligonucleotides Mediated by Cationic Lipids. Pharm. Res..

[84] Romøren K., Thu B.J., Bols N.C., Evensen Ø. (2004). Transfection efficiency and cytotoxicity of cationic liposomes in salmonid cell lines of hepatocyte and macrophage origin. Biochim. Biophys. Acta (BBA)-Biomembr..

[85] Cui S., Wang Y., Gong Y., Lin X., Zhao Y., Zhi D., Zhou Q., Zhang S. (2018). Correlation of the cytotoxic effects of cationic lipids with their headgroups. Toxicol. Res..

[86] Yang K., Lu Y., Xie F., Zou H., Fan X., Li B., Li W., Zhang W., Mei L., Feng S.-S. (2016). Cationic liposomes induce cell necrosis through lysosomal dysfunction and late-stage autophagic flux inhibition. Nanomedicine.

[87] Soenen S.J.H., Brisson A.R., De Cuyper M. (2009). Addressing the problem of cationic lipid-mediated toxicity: The magnetoliposome model. Biomaterials.

[88] Wei X., Shao B., He Z., Ye T., Luo M., Sang Y., Liang X., Wang W., Luo S., Yang S. (2015). Cationic nanocarriers induce cell necrosis through impairment of Na+/K+-ATPase and cause subsequent inflammatory response. Cell Res..

[89] Aramaki Y., Takano S., Tsuchiya S. (2001). Cationic Liposomes Induce Macrophage Apoptosis through Mitochondrial Pathway. Arch. Biochem. Biophys..

[90] Iwaoka S., Nakamura T., Takano S., Tsuchiya S., Aramaki Y. (2006). Cationic liposomes induce apoptosis through p38 MAP kinase–caspase-8–Bid pathway in macrophage-like RAW264.7 cells. J. Leukoc. Biol..

[91] Chrai S.S., Murari R., Ahmad I. (2002). Liposomes (a review): Part two: Drug delivery systems. BioPharm.

[92] Senior J.H. (1987). Fate and behavior of liposomes in vivo: A review of controlling factors. Crit. Rev. Ther. Drug Carr. Syst..

[93] Kimelberg H.K., Tracy T.F., Biddlecome S.M., Bourke R.S. (1976). The Effect of Entrapment in Liposomes on the in Vivo Distribution of [3H]Methotrexate in a Primate. Cancer Res..

[94] Yona S., Gordon S. (2015). From the Reticuloendothelial to Mononuclear Phagocyte System—The Unaccounted Years. Front. Immunol..

[95] Guilliams M., Ginhoux F., Jakubzick C., Naik S.H., Onai N., Schraml B.U., Segura E., Tussiwand R., Yona S. (2014). Dendritic cells, monocytes and macrophages: A unified nomenclature based on ontogeny. Nat. Rev. Immunol..

[96] Soundararajan A., Bao A., Phillips W.T., Perez R., Goins B.A. (2009). [(186)Re]Liposomal doxorubicin (Doxil): In vitro stability, pharmacokinetics, imaging and biodistribution in a head and neck squamous cell carcinoma xenograft model. Nucl. Med. Biol..

[97] Beyer I., Cao H., Persson J., Song H., Richter M., Feng Q., Yumul R., van Rensburg R., Li Z., Berenson R. (2012). Coadministration of epithelial junction opener JO-1 improves the efficacy and safety of chemotherapeutic drugs. Clin. Cancer Res..

[98] Arnold R.D., Mager D.E., Slack J.E., Straubinger R.M. (2005). Effect of repetitive administration of Doxorubicin-containing liposomes on plasma pharmacokinetics and drug biodistribution in a rat brain tumor model. Clin. Cancer Res..

[99] Lu W.L., Qi X.R., Zhang Q., Li R.Y., Wang G.L., Zhang R.J., Wei S.L. (2004). A pegylated liposomal platform: Pharmacokinetics, pharmacodynamics, and toxicity in mice using doxorubicin as a model drug. J. Pharm. Sci..

[100] Luo R., Li Y., He M., Zhang H., Yuan H., Johnson M., Palmisano M., Zhou S., Sun D. (2017). Distinct biodistribution of doxorubicin and the altered dispositions mediated by different liposomal formulations. Int. J. Pharm..

[101] Allen T.M., Murray L., MacKeigan S., Shah M. (1984). Chronic liposome administration in mice: Effects on reticuloendothelial function and tissue distribution. J. Pharmacol. Exp. Ther..

[102] Ellens H., Mayhew E., Rustum Y.M. (1982). Reversible depression of the reticuloendothelial system by liposomes. Biochim. Biophys. Acta (BBA)-Gen. Subj..

[103] Van Rooijen N., Sanders A. (1996). Kupffer cell depletion by liposome-delivered drugs: Comparative activity of intracellular clodronate, propamidine, and ethylenediaminetetraacetic acid. Hepatology.

[104] Storm G., ten Kate M.T., Working P.K., Bakker-Woudenberg I.A. (1998). Doxorubicin entrapped in sterically stabilized liposomes: Effects on bacterial blood clearance capacity of the mononuclear phagocyte system. Clin. Cancer Res..

[105] Rooijen N.V., Kesteren-Hendrikx E.V. (2003). “In Vivo” Depletion of Macrophages by Liposome-Mediated “Suicide”. Methods in Enzymology.

[106] Daemen T., Regts J., Meesters M., Ten Kate M.T., Bakker-Woudenberg I.A.J.M., Scherphof G.L. (1997). Toxicity of doxorubicin entrapped within long-circulating liposomes. J. Control Release.

[107] La-Beck N.M., Liu X., Wood L.M. (2019). Harnessing Liposome Interactions with the Immune System for the Next Breakthrough in Cancer Drug Delivery. Front. Pharmacol..

[108] Cruz-Leal Y., Lucatelli Laurindo M.F., Osugui L., Luzardo Mdel C., Lopez-Requena A., Alonso M.E., Alvarez C., Popi A.F., Mariano M., Perez R. (2014). Liposomes of phosphatidylcholine and cholesterol induce an M2-like macrophage phenotype reprogrammable to M1 pattern with the involvement of B-1 cells. Immunobiology.

[109] Rajan R., Sabnani M.K., Mavinkurve V., Shmeeda H., Mansouri H., Bonkoungou S., Le A.D., Wood L.M., Gabizon A.A., La-Beck N.M. (2018). Liposome-induced immunosuppression and tumor growth is mediated by macrophages and mitigated by liposome-encapsulated alendronate. J. Control. Release Off. J. Control. Release Soc..

[110] Mantovani A., Sozzani S., Locati M., Allavena P., Sica A. (2002). Macrophage polarization: Tumor-associated macrophages as a paradigm for polarized M2 mononuclear phagocytes. Trends Immunol..

[111] Miao X., Leng X., Zhang Q. (2017). The Current State of Nanoparticle-Induced Macrophage Polarization and Reprogramming Research. Int. J. Mol. Sci..

[112] Sabnani M.K., Rajan R., Rowland B., Mavinkurve V., Wood L.M., Gabizon A.A., La-Beck N.M. (2015). Liposome promotion of tumor growth is associated with angiogenesis and inhibition of antitumor immune responses. Nanomed. Nanotechnol. Biol. Med..

[113] Batenjany M.M., Boni L.T., Guo Y., Neville M.E., Bansal S., Robb R.J., Popescu M.C. (2001). The effect of cholesterol in a liposomal Muc1 vaccine. Biochim. Biophys. Acta (BBA)-Biomembr..

[114] Demel R.A., Kinsky S.C., Kinsky C.B., Van Deenen L.L.M. (1968). Effects of temperature and cholesterol on the glucose permeability of liposomes prepared with natural and synthetic lecithins. Biochim. Biophys. Acta (BBA)-Biomembr..

[115] Papahadjopoulos D., Cowden M., Kimelberg H. (1973). Role of cholesterol in membranes. Effects on phospholipid-protein interactions, membrane permeability and enzymatic activity. Biochim. Biophys. Acta.

[116] Briuglia M.L., Rotella C., McFarlane A., Lamprou D.A. (2015). Influence of cholesterol on liposome stability and on in vitro drug release. Drug Deliv. Transl. Res..

[117] Kirby C., Clarke J., Gregoriadis G. (1980). Effect of the cholesterol content of small unilamellar liposomes on their stability in vivo and In Vitro. Biochem. J..

[118] Lopez-Pinto J.M., Gonzalez-Rodriguez M.L., Rabasco A.M. (2005). Effect of cholesterol and ethanol on dermal delivery from DPPC liposomes. Int. J. Pharm..

[119] Smaby J.M., Momsen M.M., Brockman H.L., Brown R.E. (1997). Phosphatidylcholine acyl unsaturation modulates the decrease in interfacial elasticity induced by cholesterol. Biophys. J..

[120] Magarkar A., Dhawan V., Kallinteri P., Viitala T., Elmowafy M., Róg T., Bunker A. (2014). Cholesterol level affects surface charge of lipid membranes in saline solution. Sci. Rep..

[121] Papahadjopoulos D., Nir S., Ohki S. (1972). Permeability properties of phospholipid membranes: Effect of cholesterol and temperature. Biochim. Biophys. Acta (BBA)-Biomembr..

[122] Li Y., Ge M., Ciani L., Kuriakose G., Westover E.J., Dura M., Covey D.F., Freed J.H., Maxfield F.R., Lytton J. (2004). Enrichment of endoplasmic reticulum with cholesterol inhibits sarcoplasmic-endoplasmic reticulum calcium ATPase-2b activity in parallel with increased order of membrane lipids: Implications for depletion of endoplasmic reticulum calcium stores and apoptosis in cholesterol-loaded macrophages. J. Biol. Chem..

[123] Li Y., Schwabe R.F., DeVries-Seimon T., Yao P.M., Gerbod-Giannone M.C., Tall A.R., Davis R.J., Flavell R., Brenner D.A., Tabas I. (2005). Free cholesterol-loaded macrophages are an abundant source of tumor necrosis factor-alpha and interleukin-6: Model of NF-kappaB- and map kinase-dependent inflammation in advanced atherosclerosis. J. Biol. Chem..

[124] Roerdink F.H., Regts J., Handel T., Sullivan S.M., Baldeschwieler J.D., Scherphof G.L. (1989). Effect of cholesterol on the uptake and intracellular degradation of liposomes by liver and spleen; a combined biochemical and γ-ray perturbed angular correlation study. Biochim. Biophys. Acta (BBA)-Biomembr..

[125] Kaur R., Henriksen-Lacey M., Wilkhu J., Devitt A., Christensen D., Perrie Y. (2014). Effect of incorporating cholesterol into DDA:TDB liposomal adjuvants on bilayer properties, biodistribution, and immune responses. Mol. Pharm..

[126] Nakano Y., Mori M., Yamamura H., Naito S., Kato H., Taneichi M., Tanaka Y., Komuro K., Uchida T. (2002). Cholesterol inclusion in liposomes affects induction of antigen-specific IgG and IgE antibody production in mice by a surface-linked liposomal antigen. Bioconjug. Chem..

[127] Ganapathi R., Krishan A., Wodinsky I., Zubrod C.G., Lesko L.J. (1980). Effect of cholesterol content on antitumor activity and toxicity of liposome-encapsulated 1-beta-D-arabinofuranosylcytosine In Vivo. Cancer Res..

[128] Ishida T., Kojima H., Harashima H., Kiwada H. (2000). Biodistribution of liposomes and C3 fragments associated with liposomes: Evaluation of their relationship. Int. J. Pharm..

[129] Cunningham C.M., Kingzette M., Richards R.L., Alving C.R., Lint T.F., Gewurz H. (1979). Activation of Human Complement by Liposomes: A Model for Membrane Activation of the Alternative Pathway. J. Immunol..

[130] Chonn A., Cullis P.R., Devine D.V. (1991). The role of surface charge in the activation of the classical and alternative pathways of complement by liposomes. J. Immunol..

[131] Remes A., Williams D.F. (1992). Immune response in biocompatibility. Biomaterials.

[132] Lynn A.D., Blakney A.K., Kyriakides T.R., Bryant S.J. (2011). Temporal progression of the host response to implanted poly(ethylene glycol)-based hydrogels. J. Biomed. Mater. Res. A.

[133] Szebeni J., Moghimi S.M. (2009). Liposome triggering of innate immune responses: A perspective on benefits and adverse reactions. J. Liposome Res..

[134] Dobrovolskaia M.A., Aggarwal P., Hall J.B., McNeil S.E. (2008). Preclinical studies to understand nanoparticle interaction with the immune system and its potential effects on nanoparticle biodistribution. Mol. Pharm..

[135] Dobrovolskaia M.A., McNeil S.E. (2007). Immunological properties of engineered nanomaterials. Nat. Nanotechnol..

[136] Lee Y.K., Choi E.-J., Webster T.J., Kim S.-H., Khang D. (2014). Effect of the protein corona on nanoparticles for modulating cytotoxicity and immunotoxicity. Int. J. Nanomed..

[137] Kinsky S.C., Haxby J.A., Zopf D.A., Alving C.R., Kinsky C.B. (1969). Complement-dependent damage to liposomes prepared from pure lipids and Forssman hapten. Biochemistry.

[138] Wang X., Ishida T., Kiwada H. (2007). Anti-PEG IgM elicited by injection of liposomes is involved in the enhanced blood clearance of a subsequent dose of PEGylated liposomes. J. Control Release.

[139] Alving C.R., Rao M., Steers N.J., Matyas G.R., Mayorov A.V. (2012). Liposomes containing lipid A: An effective, safe, generic adjuvant system for synthetic vaccines. Expert Rev. Vaccines.

[140] Alving C.R. (1991). Liposomes as carriers of antigens and adjuvants. J. Immunol. Methods.

[141] Perrie Y., Crofts F., Devitt A., Griffiths H.R., Kastner E., Nadella V. (2016). Designing liposomal adjuvants for the next generation of vaccines. Adv. Drug Deliv. Rev..

[142] Didierlaurent A.M., Laupeze B., Di Pasquale A., Hergli N., Collignon C., Garcon N. (2017). Adjuvant system AS01: Helping to overcome the challenges of modern vaccines. Expert Rev. Vaccines.

[143] Badiee A., Jaafari M.R., Khamesipour A., Samiei A., Soroush D., Kheiri M.T., Barkhordari F., McMaster W.R., Mahboudi F. (2009). The role of liposome charge on immune response generated in BALB/c mice immunized with recombinant major surface glycoprotein of Leishmania (rgp63). Exp. Parasitol..

[144] Badiee A., Khamesipour A., Samiei A., Soroush D., Shargh V.H., Kheiri M.T., Barkhordari F., Robert Mc Master W., Mahboudi F., Jaafari M.R. (2012). The role of liposome size on the type of immune response induced in BALB/c mice against leishmaniasis: rgp63 as a model antigen. Exp. Parasitol..

[145] Henriksen-Lacey M., Devitt A., Perrie Y. (2011). The vesicle size of DDA:TDB liposomal adjuvants plays a role in the cell-mediated immune response but has no significant effect on antibody production. J. Control Release.

[146] Yamamoto S., Ishida T., Inoue A., Mikami J., Muraguchi M., Ohmoto Y., Kiwada H. (2002). HEPC-based liposomes trigger cytokine release from peripheral blood cells: Effects of liposomal size, dose and lipid composition. Int. J. Pharm..

[147] Niwa K., Ikebuchi K., Fujihara M., Abe H., Wakamoto S., Ito T., Yamaguchi M., Sekiguchi S. (1998). Inflammatory cytokine production in whole blood modified by liposome-encapsulated hemoglobin. Artif. Cells Blood Substit. Immobil. Biotechnol..

[148] Noris M., Remuzzi G. (2013). Overview of complement activation and regulation. Semin. Nephrol..

[149] van den Berg R.H., Faber-Krol M.C., Sim R.B., Daha M.R. (1998). The First Subcomponent of Complement, C1q, Triggers the Production of IL-8, IL-6, and Monocyte Chemoattractant Peptide-1 by Human Umbilical Vein Endothelial Cells. J. Immunol..

[150] Wagner H., Dixon F.J. (1999). Bacterial CpG DNA Activates Immune Cells to Signal Infectious Danger. Advances in Immunology.

[151] Ahmad-Nejad P., Hacker H., Rutz M., Bauer S., Vabulas R.M., Wagner H. (2002). Bacterial CpG-DNA and lipopolysaccharides activate Toll-like receptors at distinct cellular compartments. Eur. J. Immunol..

[152] Sellins K., Fradkin L., Liggitt D., Dow S. (2005). Type I interferons potently suppress gene expression following gene delivery using liposome(-)DNA complexes. Mol. Ther..

[153] Yasuda S., Yoshida H., Nishikawa M., Takakura Y. (2010). Comparison of the type of liposome involving cytokine production induced by non-CpG Lipoplex in macrophages. Mol. Pharm..

[154] Pusztai L., Mendoza T.R., Reuben J.M., Martinez M.M., Willey J.S., Lara J., Syed A., Fritsche H.A., Bruera E., Booser D. (2004). Changes in plasma levels of inflammatory cytokines in response to paclitaxel chemotherapy. Cytokine.

[155] Szebeni J. (1998). The interaction of liposomes with the complement system. Crit Rev. Ther. Drug Carr. Syst..

[156] Cullis P.R., Chonn A., Semple S.C. (1998). Interactions of liposomes and lipid-based carrier systems with blood proteins: Relation to clearance behaviour In Vivo. Adv. Drug Deliv. Rev..

[157] Bradley A.J., Brooks D.E., Norris-Jones R., Devine D.V. (1999). C1q Binding to liposomes is surface charge dependent and is inhibited by peptides consisting of residues 14–26 of the human C1qA chain in a sequence independent manner. Biochim. Biophys. Acta (BBA)-Biomembr..

[158] Harashima H., Sakata K., Funato K., Kiwada H. (1994). Enhanced Hepatic Uptake of Liposomes Through Complement Activation Depending on the Size of Liposomes. Pharm. Res..

[159] Liu Z.-Y., Solow R., Hu V.W. (1988). Fluorescence analysis of size distribution and mode of dye release from carboxyfluorescein-loaded vesicles: Application to the study of complement-membrane interactions. Biochim. Biophys. Acta (BBA)-Biomembr..

[160] Alving C.R., Richards R.L., Guirguis A.A. (1977). Cholesterol-Dependent Human Complement Activation Resulting in Damage to Liposomal Model Membranes. J. Immunol..

[161] Szebeni J., Baranyi L., Savay S., Milosevits J., Bunger R., Laverman P., Metselaar J.M., Storm G., Chanan-Khan A., Liebes L. (2002). Role of complement activation in hypersensitivity reactions to doxil and hynic PEG liposomes: Experimental and clinical studies. J. Liposome Res..

[162] Chanan-Khan A., Szebeni J., Savay S., Liebes L., Rafique N.M., Alving C.R., Muggia F.M. (2003). Complement activation following first exposure to pegylated liposomal doxorubicin (Doxil): Possible role in hypersensitivity reactions. Ann. Oncol..

[163] van den Hoven J.M., Nemes R., Metselaar J.M., Nuijen B., Beijnen J.H., Storm G., Szebeni J. (2013). Complement activation by PEGylated liposomes containing prednisolone. Eur. J. Pharm. Sci..

[164] Fülöp T., Kozma G.T., Vashegyi I., Mészáros T., Rosivall L., Urbanics R., Storm G., Metselaar J.M., Szebeni J. (2019). Liposome-induced hypersensitivity reactions: Risk reduction by design of safe infusion protocols in pigs. J. Control Release.

[165] Szebeni J., Baranyi L., Savay S., Bodo M., Morse D.S., Basta M., Stahl G.L., Bunger R., Alving C.R. (2000). Liposome-induced pulmonary hypertension: Properties and mechanism of a complement-mediated pseudoallergic reaction. Am. J. Physiol. Heart Circ. Physiol..

[166] Ingen-Housz-Oro S., Pham-Ledard A., Brice P., Lebrun-Vignes B., Zehou O., Reitter D., Ram-Wolff C., Dupin N., Bagot M., Chosidow O. (2017). Immediate hypersensitivity reaction to pegylated liposomal doxorubicin: Management and outcome in four patients. Eur. J. Derm..

[167] Arning M., Heer-Sonderhoff A.H., Wehmeier A., Schneider W. (1995). Pulmonary toxicity during infusion of liposomal amphotericin B in two patients with acute leukemia. Eur. J. Clin. Microbiol. Infect. Dis..

[168] Judge A., McClintock K., Phelps J.R., Maclachlan I. (2006). Hypersensitivity and loss of disease site targeting caused by antibody responses to PEGylated liposomes. Mol. Ther..

[169] Ringden O., Andstrom E., Remberger M., Svahn B.M., Tollemar J. (1994). Allergic reactions and other rare side-effects of liposomal amphotericin. Lancet.

[170] Alberts D.S., Garcia D.J. (1997). Safety Aspects of Pegylated Liposomal Doxorubicin in Patients with Cancer. Drugs.

[171] Szebeni J., Fontana J.L., Wassef N.M., Mongan P.D., Morse D.S., Dobbins D.E., Stahl G.L., Bunger R., Alving C.R. (1999). Hemodynamic changes induced by liposomes and liposome-encapsulated hemoglobin in pigs: A model for pseudoallergic cardiopulmonary reactions to liposomes. Role of complement and inhibition by soluble CR1 and anti-C5a antibody. Circulation.

[172] Hammerschmidt D., Hudson L., Weaver L.J., Craddock P., Jacob H. (1980). Association of Complement Activation and Elevated Plasma-C5a with Adult Respiratory Distress Syndrome: Pathophysiological Relevance and Possible Prognostic Value. Lancet.

[173] Marceau F., Lundberg C., Hugli T.E. (1987). Effects of the anaphylatoxins on circulation. Immunopharmacology.

[174] Kozma G.T., Mészáros T., Vashegyi I., Fülöp T., Örfi E., Dézsi L., Rosivall L., Bavli Y., Urbanics R., Mollnes T.E. (2019). Pseudo-anaphylaxis to Polyethylene Glycol (PEG)-Coated Liposomes: Roles of Anti-PEG IgM and Complement Activation in a Porcine Model of Human Infusion Reactions. ACS Nano.

[175] Szebeni J., Muggia F., Gabizon A., Barenholz Y. (2011). Activation of complement by therapeutic liposomes and other lipid excipient-based therapeutic products: Prediction and prevention. Adv. Drug Deliv. Rev..

[176] Weiss R.B., Donehower R.C., Wiernik P.H., Ohnuma T., Gralla R.J., Trump D.L., Baker J.R., Van Echo D.A., Von Hoff D.D., Leyland-Jones B. (1990). Hypersensitivity reactions from taxol. J. Clin. Oncol..

[177] Castells M.C., Tennant N.M., Sloane D.E., Hsu F.I., Barrett N.A., Hong D.I., Laidlaw T.M., Legere H.J., Nallamshetty S.N., Palis R.I. (2008). Hypersensitivity reactions to chemotherapy: Outcomes and safety of rapid desensitization in 413 cases. J. Allergy Clin. Immunol..

[178] Szebeni J., Bedőcs P., Rozsnyay Z., Weiszhár Z., Urbanics R., Rosivall L., Cohen R., Garbuzenko O., Báthori G., Tóth M. (2012). Liposome-induced complement activation and related cardiopulmonary distress in pigs: Factors promoting reactogenicity of Doxil and AmBisome. Nanomed. Nanotechnol. Biol. Med..

[179] (2000). ALZA Pharmaceuticals.

[180] Szebeni J., Simberg D., González-Fernández Á., Barenholz Y., Dobrovolskaia M.A. (2018). Roadmap and strategy for overcoming infusion reactions to nanomedicines. Nat. Nanotechnol..

[181] Ishida T., Wang X., Shimizu T., Nawata K., Kiwada H. (2007). PEGylated liposomes elicit an anti-PEG IgM response in a T cell-independent manner. J. Control Release.

[182] Verhoef J.J.F., Anchordoquy T.J. (2013). Questioning the Use of PEGylation for Drug Delivery. Drug Deliv. Transl. Res..

[183] Ichihara M., Shimizu T., Imoto A., Hashiguchi Y., Uehara Y., Ishida T., Kiwada H. (2010). Anti-PEG IgM Response against PEGylated Liposomes in Mice and Rats. Pharmaceutics.

[184] Mohamed M., Abu Lila A.S., Shimizu T., Alaaeldin E., Hussein A., Sarhan H.A., Szebeni J., Ishida T. (2019). PEGylated liposomes: Immunological responses. Sci. Technol. Adv. Mater..

[185] Yang Q., Lai S.K. (2015). Anti-PEG immunity: Emergence, characteristics, and unaddressed questions. Wiley Interdiscip. Rev. Nanomed. Nanobiotechnol..

[186] Mond J.J., Vos Q., Lees A., Snapper C.M. (1995). T cell independent antigens. Curr. Opin. Immunol..

[187] Boes M. (2000). Role of natural and immune IgM antibodies in immune responses. Mol. Immunol..

[188] Ishida T., Ichihara M., Wang X., Kiwada H. (2006). Spleen plays an important role in the induction of accelerated blood clearance of PEGylated liposomes. J. Control Release.

[189] Bucke W.E., Leitzke S., Diederichs J.E., Borner K., Hahn H., Ehlers S., Müller R.H. (1998). Surface-Modified Amikacin-Liposomes: Organ Distribution and Interaction with Plasma Proteins. J. Drug Target..

[190] Cheng T.-L., Wu P.-Y., Wu M.-F., Chern J.-W., Roffler S.R. (1999). Accelerated Clearance of Polyethylene Glycol-Modified Proteins by Anti-Polyethylene Glycol IgM. Bioconjug. Chem..

[191] Hauet T., Eugene M. (2008). A new approach in organ preservation: Potential role of new polymers. Kidney Int..

[192] Dams E.T.M., Laverman P., Oyen W.J.G., Storm G., Scherphof G.L., van der Meer J.W.M., Corstens F.H.M., Boerman O.C. (2000). Accelerated Blood Clearance and Altered Biodistribution of Repeated Injections of Sterically Stabilized Liposomes. J. Pharmacol. Exp. Ther..

[193] Laverman P., Carstens M.G., Boerman O.C., Dams E.T.M., Oyen W.J.G., van Rooijen N., Corstens F.H.M., Storm G. (2001). Factors Affecting the Accelerated Blood Clearance of Polyethylene Glycol-Liposomes upon Repeated Injection. J. Pharmacol. Exp. Ther..

[194] Ishida T., Maeda R., Ichihara M., Irimura K., Kiwada H. (2003). Accelerated clearance of PEGylated liposomes in rats after repeated injections. J. Control Release.

[195] Xu H., Ye F., Hu M., Yin P., Zhang W., Li Y., Yu X., Deng Y. (2015). Influence of phospholipid types and animal models on the accelerated blood clearance phenomenon of PEGylated liposomes upon repeated injection. Drug Deliv..

[196] Ishida T., Ichikawa T., Ichihara M., Sadzuka Y., Kiwada H. (2004). Effect of the physicochemical properties of initially injected liposomes on the clearance of subsequently injected PEGylated liposomes in mice. J. Control Release.

[197] Semple S.C., Harasym T.O., Clow K.A., Ansell S.M., Klimuk S.K., Hope M.J. (2005). Immunogenicity and Rapid Blood Clearance of Liposomes Containing Polyethylene Glycol-Lipid Conjugates and Nucleic Acid. J. Pharmacol. Exp. Ther..

[198] Ishida T., Atobe K., Wang X., Kiwada H. (2006). Accelerated blood clearance of PEGylated liposomes upon repeated injections: Effect of doxorubicin-encapsulation and high-dose first injection. J. Control Release.

[199] Ishida T., Kashima S., Kiwada H. (2008). The contribution of phagocytic activity of liver macrophages to the accelerated blood clearance (ABC) phenomenon of PEGylated liposomes in rats. J. Control Release.

[200] Koide H., Asai T., Hatanaka K., Akai S., Ishii T., Kenjo E., Ishida T., Kiwada H., Tsukada H., Oku N. (2010). T cell-independent B cell response is responsible for ABC phenomenon induced by repeated injection of PEGylated liposomes. Int. J. Pharm..

[201] Suzuki T., Ichihara M., Hyodo K., Yamamoto E., Ishida T., Kiwada H., Ishihara H., Kikuchi H. (2012). Accelerated blood clearance of PEGylated liposomes containing doxorubicin upon repeated administration to dogs. Int. J. Pharm..

[202] Lopes T.C.M., Silva D.F., Costa W.C., Frézard F., Barichello J.M., Silva-Barcellos N.M., de Lima W.G., Rezende S.A. (2018). Accelerated Blood Clearance (ABC) Phenomenon Favors the Accumulation of Tartar Emetic in Pegylated Liposomes in BALB/c Mice Liver. Pathol. Res. Int..

[203] Wang F.L., Wang H.H., Wu Y.F., Wang L., Zhang L., Ye X., Peng D.Y., Chen W.D. (2019). Activation of PXR-Cytochrome P450s axis: A Possible Reason for the Enhanced Accelerated Blood Clearance Phenomenon of PEGylated Liposomes In Vivo. Drug Metab. Dispos..

[204] Ishida T., Kiwada H. (2008). Accelerated blood clearance (ABC) phenomenon upon repeated injection of PEGylated liposomes. Int. J. Pharm..

[205] Gabizon A., Isacson R., Rosengarten O., Tzemach D., Shmeeda H., Sapir R. (2008). An open-label study to evaluate dose and cycle dependence of the pharmacokinetics of pegylated liposomal doxorubicin. Cancer Chemother. Pharm..

[206] Yang Q., Jacobs T.M., McCallen J.D., Moore D.T., Huckaby J.T., Edelstein J.N., Lai S.K. (2016). Analysis of Pre-existing IgG and IgM Antibodies against Polyethylene Glycol (PEG) in the General Population. Anal. Chem..

[207] Wang F., Ye X., Wu Y., Wang H., Sheng C., Peng D., Chen W. (2019). Time Interval of Two Injections and First-Dose Dependent of Accelerated Blood Clearance Phenomenon Induced by PEGylated Liposomal Gambogenic Acid: The Contribution of PEG-Specific IgM. J. Pharm. Sci..

